# Identifying communicative functions in discourse with content types

**DOI:** 10.1007/s10579-021-09554-4

**Published:** 2021-08-04

**Authors:** Tommaso Caselli, Rachele Sprugnoli, Giovanni Moretti

**Affiliations:** 1 Rijksuniversiteit Groningen, Groningen, Netherlands; 2 Università Cattolica del Sacro Cuore, Milan, Italy

**Keywords:** Content types, Corpus annotation, Neural networks, Across genre, Across time

## Abstract

Texts are not monolithic entities but rather coherent collections of micro illocutionary acts which help to convey a unitary message of content and purpose. Identifying such text segments is challenging because they require a fine-grained level of analysis even within a single sentence. At the same time, accessing them facilitates the analysis of the communicative functions of a text as well as the identification of relevant information. We propose an empirical framework for modelling micro illocutionary acts at clause level, that we call content types, grounded on linguistic theories of text types, in particular on the framework proposed by Werlich in 1976. We make available a newly annotated corpus of 279 documents (for a total of more than 180,000 tokens) belonging to different genres and temporal periods, based on a dedicated annotation scheme. We obtain an average Cohen’s kappa of 0.89 at token level. We achieve an average F1 score of 74.99% on the automatic classification of content types using a bi-LSTM model. Similar results are obtained on contemporary and historical documents, while performances on genres are more varied. This work promotes a discourse-oriented approach to information extraction and cross-fertilisation across disciplines through a computationally-aided linguistic analysis.

## Introduction

Texts are complex linguistic entities. The intended purpose of a text (e.g., to inform, to entertain, to convince, among others) is an essential aspect that shapes them. Purpose drives how the logical structure of a document, i.e. its hierarchical arrangement in sections, paragraphs, sentences and the like, is organised. This structure is reflected in the functional organisation of the information flow, creating expectations on where the desired information may be located. Texts, thus, become instances of a particular class of discourse, normally defined as genres (Biber, 1989). Texts of the same genre are assumed to play the same role in communication, for instance, a newspaper article is assumed to be informative rather than descriptive. The way this is achieved is through sets of conventionalised text segments that specifically contribute to the associated communicative function. We call these segments *content types* (CTs).

Segmenting a text into CTs facilitates the analysis of both the organisation and flow of information. For instance, a writer will present major events in narrative segments, provide opinions and evaluations with arguments, and details in descriptions. At the same time, narrative passages will facilitate the connection between events and, thus, express the progress of a story; argumentative segments will aim at convincing the reader of an opinion; instructive passages will be reserved to express instructions. This potentially opens up to their application in numerous downstream tasks such as Event Detection (Choubey et al., 2020), Summarisation (Liakata et al., 2012; Teufel & Moens, 2002), Genre Identification (Worsham & Kalita, 2018), and Essay Scoring (Song et al., 2017), among others.

The definition and identification of CTs is, nevertheless, challenging. First, theoretical frameworks have targeted this topic from different perspectives ranging from cognitive science to theoretical linguistics, giving rise to fragmented, and sometimes incompatible, labelling systems and levels of analysis. Second, related work in Computational Linguistics (CL) and Natural Language Processing (NLP), on the one hand, mirrors this fragmented scenario, and, on the other hand, it introduces extra complexity as new frameworks are proposed and developed. Third, annotated corpora, with very few exceptions (Mavridou et al., 2015), have been mainly conducted on specific domains like literary texts or semi-structured documents, like scientific papers or narrative essays, failing to systematically apply the proposed frameworks across genres. Finally, no investigation has taken into account time as a possible variable that may impact the presence and distribution of such segments in documents.

Given these premises, we formulate the following research questions:

- How can we translate existing theories into an empirical framework for the annotation of CTs across genres and temporal periods?
- What is the impact of different word embedding representations for the automatic classification of CTs? Furthermore, what is the lower bound of the best model with automatically segmented data?
- What is the impact of changes in text genres and/or time for the portability of trained models?

In this article, we push forward the state of the art in computational analysis of coarse-grained discourse segmentation by tackling the identified challenges and issues. We promote a vision of texts as coherent collections of micro illocutionary acts: a mixture of narrations, descriptions, and opinions, among others, that tend to co-occur, each contributing to the composition and development of the message and purpose encoded into the discourse.

### Our contribution

We summarise our contributions as follows:

- we create a **new corpus** of texts from different genres and temporal periods manually annotated with CTs, corresponding to clauses with specific semantic and functional characteristics, that facilitate the analysis of texts as a composition of units. The corpus contains 279 documents comprising more than 180,000 tokens and 20,000 clauses (Sect. 4);
- we run an extensive set of **experiments** on the automatic classification of CTs (Sect. 5.1) and the impact of clause extent prediction (Sect. 5.3);
- we asses the impact of changes in text genres and time for the **portability** of trained models for CTs. (Sect. 5.4).

This work builds on Sprugnoli et al. (2017a) where we first tested our annotation scheme and run preliminary experiments using linear models. The new corpus, the annotation guidelines, the data statement (Bender & Friedman 2018), as well as the best performing model are freely available on GitHub (https://github.com/johnnymoretti/ContentTypes) and on Dataverse.NL (https://doi.org/10.34894/TYB4PF).

## Theoretical background

Our work on CTs is grounded on linguistic theories of text types. In literature, the notion of text types often overlaps with the notions of genre, register, and discourse modes. Consensus on their classification among scholars is still lacking. Although a perfect overlap across the different typologies proposed in literature is not possible, Table 1 attempts to systematically organise the text type labels and their correspondences.^1^

Kinneavy (1971) defines four text types as cognitive categories expressing the way in which reality is viewed, using the basic distinctions between static and dynamic, and between individual and collective.

**Table 1:** Summary of the labels and most likely correspondences for text types classification among the reviewed works

Kinneavy (1971)	Werlich (1976)	Beaugrande and Dressler (1981)	Longacre (1983)	Adam (1985)	Biber (1989)	Virtanen (1992a)	Fludernik (2000)	Smith (2003)
Narration	Narrative	Narrative	Narrative	Narrative	Imaginative narrative	Narrative	Narrative	Narrative
					General narrative exposition			
Description	Descriptive	Descriptive	–	Description	–	Description	–	Description
Evaluation	Argumentative	Argumentative	–	Argumentative	–	Argument	Argumentative	Argument
Classification	Expository	Didactic	Expository	Explicative	Scientific exposition	Exposition	–	Informative
		Scientific			Learned exposition			
–	Instructive	–	Procedural	Injunctive	–	Instruction	Instructive	–
–	–	–	Behavioral	–	–	–	–	–
–	–	–	–	Conversational	Interpersonal interaction	–	Conversational	–
					Informational interaction			
–	–	Literary	–	Rhetorical		–	Reflective	–
		Poetic						
–	–	–	–	Predictive		–	–	–
–	–	–	–	–	Situated reporting	–	–	Report
–	–	–	–	–	Involved persuasion	–	–	–

Werlich (1976) also adopts a cognitive approach: five cognitive properties of the text (i.e., perception in time, perception in space, comprehension of general concepts, judging and planning) characterise five text types. Werlich adds a specific type for texts that aim to guide the reader in performing a task (i.e., instructive) and this differs with respect to the Kinneavy’s model. Werlich’s classification can be approached at three different levels: as abstract prototypical types, as specific text forms, and as actual linguistic realisations. At this latter level, types are defined by aligning textual functions with surface text structures at paragraph or sentence levels.

Beaugrande and Dressler (1981) propose seven text types (i.e., narrative, descriptive, argumentative, scientific, didactic, literary, and poetic). These categories apply at document level. On the contrary, they do not recognise an autonomous status to the instructive text type while providing a finer-grained characterisation of the exposition type with a distinction between didactic and scientific texts.

Longacre (1983) focuses on ideal text types, called “deep structure genres”

and uses the label procedural instead of instructive; he also eliminates both the descriptive and the argumentative text types.^2^ Longacre (1983)’s model gives emphasis to the oral mode of communication through the introduction of the behavioural text type. Similarly, Adam (1985) focuses his work on the global and deep structures of text and takes into consideration the oral dimension. He expands Werlich (1976)’s scheme trying to connect each text type to at least one speech act (Searle, 1969).

Virtanen and Wârvik (1987) try to combine the models by Kinneavy, Werlich, Longacre and Adam proposing a scheme based on seven different levels. In particular, they add two extra levels on top of Werlich’s tripartite proposal: one corresponding to cognitive processes and another representing communicative functions (Jakobson, 1960).

A different approach, based on empirical observations and statistical measures, is followed by Biber (1989) who develops his text typology looking at the frequency of occurrence of 67 linguistic features within a corpus of 481 documents. The resulting text types are conceived as a “grouping of texts [documents] that are markedly similar to one another with respect to their dimension characterizations.” (Biber, 1989, p. 3)

In a later proposal, Virtanen (1992b) simplifies the original seven layer model (Virtanen & Wârvik, 1987): she also directly formulates a list of text types, called “discourse types”, and frames them as functions of discourse connected to cognitive processes (Tsiplakou & Floros, 2013). In a similar way, Fludernik (2000) proposes a three-level scheme in which text types are defined on the basis of communicative functions at a general level called *macrogenres*: this level is placed above both the level of genre (e.g., guidebooks, letters) and the level of actual linguistic realisations. As Longacre, Fludernik disputes the existence of a descriptive text type because there are only few texts having a description as the only discourse strategy. Moreover, the author inherits the conversational and rhetorical types from Adam’s model but uses a different label for the latter (i.e., reflective) to clarify its meta-linguistic nature. Following Fludernik (2000)’s terminology, the surface level of text can be classified into different *discourse modes*. This expression is further adopted by Smith (2003) to identify five different types of textual passages characterised by specific linguistic forms and pragmatic structures.

As it emerges from this overview, only the narrative text type is present in all typologies, and approaches for the definition of text types vary from cognitive to functional, from purely theoretical to empirical and corpus-based. Other differences concern the number of types, the terminology, and the range of texts taken into consideration (written/oral). Further differences affect the application of these frameworks to documents: in some cases there are no explicit instructions as the interest is on a more abstract formalisation (Adam, 1985; Beaugrande & Dressler, 1981; Longacre, 2013), while in other cases differences affect the granularity of the units of analysis, ranging between segments (e.g. paragraphs or passages) (Smith, 2001) to sentences (Virtanen, 1992b; Werlich, 1976).

In our work, we adopt Werlich’s modelisation because in his proposal surface text realisations are aligned with textual functions, identifying a dimension of variation internal to the text rather than pointing to the writer’s purpose and topics (Biber, 1989; Fludernik, 2000). Werlich explicitly indicates such linguistic features for each text types, based on Quirk (1972)’s grammar. For example, imperatives are a feature of instructive sentences, temporal markers are typical of narrative sentences and location markers characterise descriptive sentences. In addition, he identifies six “phenomenon sentences” peculiar of the five text types. For example, the **subject-predicate-adverb_of_place** structure is widespread in descriptions (Santini, 2005). The attention to linguistic features and to surface structures together with the alignment of these structures with textual functions facilitates the analysis of texts on a sentence-by-sentence level, or even into smaller units (such as clauses).

## Related work in computational linguistics

Text types represent a coarse level of analysis of discourse when compared to other areas of work such as *coherence relations* (Asher and Lascarides, 1994; Cristea et al., 1998; Grosz & Sidner, 1986; Hobbs, 1985; Mann & Thompson, 1988; Prasad et al., 2008). The study of coherence relations can be roughly summarised as understanding the inner logic behind “the linear order of sentences” (Stede, 2011, p. 79). The focus is on the identification of *hierarchical structures* (both at local and at global levels) that compose the coherence of discourse and allow access to the discourse entities. On the other hand, text types and their associated frameworks investigate the structure of the document’s content as it may be induced by the type of the text itself, or its genre according to some terminologies.

CT’s framework can be associated with this flat analysis of discourse but it presents points of divergence and advantages. In particular, the proposed framework identifies categories that are independent of specific text types, or genres. For instance, a CT label is not proposed because of the text type of the document (e.g., a travel report) but rather it is an abstraction that encompasses differences across text types. Moreover, CTs follow a linear interpretation of the structure of the text. This results in a simpler representation when compared to building complex semantic relational structures.

Focusing on text types, related work in Computational Linguistics (CL) and Natural Language Processing (NLP) represents a nice example of how theoretical frameworks are translated into computational models. We structure the remainder of this section into two sub-categories: *communicative function* frameworks and *genre-specific* frameworks as they are the closest and most relevant to this article’s contribution.

### Communicative function frameworks

Communicative frameworks qualify as the computational counterparts of the frameworks and theories illustrated in Sect. 2. All of these approaches remove the genre constraint while capturing more general communicative functions in the structure of the documents. This is a point of connection and similarity with our proposed empirical framework. However, we assume that the identification of content types primarily resides in the meaning, i.e., the content of the text passage in analysis, rather than only in its communicative function. A further difference concerns the types of text and the time periods. While rich, previous work has focused on the analysis of one text type at the time and without considering differences across time periods. Our work moves away from these aspects by considering multiple text types and time periods.

Cocco et al. (2011) apply an annotation scheme for types of discourse to a corpus of three French short stories of the XIX century. They identify six discourse types (Narrative, Argumentative, Descriptive, Explicative, Dialoged, and Injunctive) by merging together linguistics and psycho-linguistic frameworks (Adam, 2011; Bronckart, 1997). Their annotation applies at clause level, because sentences were not sufficiently fine-grained for the proposed analysis. The corpus contains 905 clauses and 7,525 tokens. Automatic identification of text types is done using a clustering approach.

Song et al. (2017) present a neural sequence labelling model based on Gated Recurrent Units (GRU) to automatically identify discourse modes in narrative essays written by Chinese students in native language. The corpus is composed by 415 narrative essays, with 32 sentences and 670 on average. Their annotation is based on an adapted version of Smith (2003)’s model and it is composed by five classes (Narration, Description, Exposition, Argument, and Emotion Expressing). Sentences are the annotation units, like in Argumentative Zoning and Core Scientific Concept, but annotators may assign multiple labels in case of ambiguities. The inter-annotator agreement based on Cohen’s K is .72. Their system obtains a global F-measure of 70.0%, with values for each class ranging between 48.3% (for Argument) and 81.5% (for Narration).

A recent work in the area of Literary Studies in German (Schlör et al., 2019) proposes a sentence-based annotation scheme using only three discourse types (Narrative, Descriptive, and Argumentative). The authors generate two data sets according to the annotators’ agreement: one of 883 sentences , DS, where all annotators agree; and a second of 1503 sentences, DM, where labels are assigned on the basis of a majority voting. Experiments compared performances of a linear SVM (Support-Vector Machines) against an modified version of the network model from Song et al. (2017), with the SVM outperforming the GRU model (averaged accuracy of 86.4% *vs.* 80.1%).

Finally, Banisakher et al. (2020) develop a classifier for the identification of the communicative function of paragraphs in news articles in English on the basis of Van Dijk (1988)’s hierarchical theory of news discourse. The corpus has 50 documents, for a total of 28,236 words divided in 644 paragraphs. The proposed annotation characterises how individual paragraphs convey information about the events in the narrative development of the article. The authors obtain an average F1 score of 71.0%, beating previous a previous approach based on SVM  (Yarlott et al., 2018).

### Genre-specific frameworks

Genre-specific frameworks assume that each text belonging to a specific genre is composed by predefined passages with dedicated rhetorical functions. Two major genre-specific frameworks are Argumentative Zoning (AZ) (Teufel & Moens, 2002; Teufel et al., 2009) and Core Scientific Concept (CoreSC) (Liakata et al., 2010). Both frameworks have been developed for classifying scientific texts over which they have slightly different, though complementary, perspectives.

CTs differ from AZ, CoreSC, and related approaches as they are genre-independent and express broader and more general *functions* of communication. No assumption is done on the presence or absence of specific rhetorical passages according to the assumed type of text or genre. In our case, changing genre does not require changing the class naming or their granularity.

*Argumentative Zoning* AZ assumes that scientific articles are structured around the notion of *knowledge claims*, according to which writing a scientific paper is *claiming ownership for new knowledge to be integrated into the scientific repository of a discipline and convince the audience/reviewers of the validity of the claims*. Knowledge claims are realised in specific text blocks, called *zones*, expressing rhetorical functions in terms of problem solving, intellectual attribution, and relatedness among articles. The original AZ scheme contains 7 classes (Aim, Textual, Own, Background, Contrast, Basis, Other) further extended to 15 (Liakata et al., 2010; Teufel et al., 2009). The original AZ corpus consists of 80 CL conference articles (12,188 sentences; 285,934 words). The revised version with 15 classes, AZ-II (Teufel et al., 2009), consists of 30 chemistry papers (3745 sentences, 3650 words on average per article) and 9 CL papers (1629 sentences, 4219 words on average per paper). AZ has sentences as its minimal annotation unit and has been applied to several domains such as Biology (Mizuta et al., 2006), Law (Hachey & Grover, 2006), and Bio-medicine (Guo et al., 2010). This, however, required changes in the class naming and granularity to meet the specific characteristics of a discipline, as in the case of Biology and Law, or to apply the scheme to a specific section of the article, such as abstracts. For instance, Mizuta et al. (2006) introduced the labels connection (CNN) and difference (DFF) as a domain-specific adjustment to reflect the established methodology in the domain of biology where “the focus is on a more neutral comparison between the author’s data/findings and those by others.” (Mizuta et al., 2006, p. 471).

Automatic identification of AZ has been mainly based on supervised, feature-based, linear models, using SVM (Guo et al., 2010), naive Bayes (Guo et al., 2010; Teufel & Moens, 2002), or Conditional Random Fields (CRF) (Guo et al., 2010). Results on AZ experiments are usually reported using 10-fold cross-validation, due to limited size of the annotated corpora with F-measure values that vary a lot from class to class (e.g., 26.0% (Contrast), 52.0% (Aim), and up to 86.0% (Own) (Teufel & Moens, 2002)).

*Core Scientific Concept* The CoreSC framework interprets scientific papers (or abstracts (Guo et al., 2010)) as *human-readable interpretations of a scientific investigation*. The proposed annotation aims at marking-up the passages expressing the *components* of a research rather than their rhetorical or communicative functions. CoreSC is ontology-motivated, it applies to sentences, and it is structured along three layers. The first layer is composed by 11 categories (i.e., Motivation, Goal, Object, Method, Experiment, Observation, Result, Conclusion, Hypothesis, Model, and Background) that express constitutive and indispensable concepts to conduct a scientific investigation; the second layer highlights properties of the concepts such as the novelty of a method; finally, the third layer establishes coreferential relations across instances of the same concept (as annotated from the first layer). The CoreSC corpus is composed by 265 papers from Chemistry and Bio-chemistry. The annotation, conducted using domain experts, is carried on incrementally and independently from the CoreSC class assigned to the previous sentence. Experiments on automatic CoreSC identification (Liakata et al., 2012) framed the task both as a text classification problem, using a linear SVM, and as a sequence labelling one, using a CRF. Evaluation, based on a 10-fold cross-validation, registered an F-measure (micro-averaged) ranging between 18.0% (Motivation) and 76.0% (Experiment).

Complementarity between the AZ framework and the CoreSC scheme has been assessed and measured by means of the Goodman-Kruskal lambda L statistic (Liakata et al., 2010), suggesting that their combination would be beneficial for the analysis of texts. In particular, Guo et al. (2010) show that the use of AZ classes as the independent variable to predict CoreCS would lead to 38% error reduction while, vice-versa, the error reduction in AZ classes would be of 35%.

Recently, Huang et al. (2020) applied an independently developed annotation scheme, SOLVENT (Chan et al., 2018), to identify aspects of research papers to the abstracts of the CODA-19^3^ collection using crowdsourcing. They report an overall accuracy on automatic label classification of 77.4% using SciBERT.

## Annotation study and corpus creation

This section illustrates the process of data selection, annotation, curation, and evaluation employed for the creation of the new corpus of CTs. Given that the study of CTs is fragmented, with the existence of different annotation schemes, mainly dependent on the document genre, and with a lack of established evaluation procedures, we aim at keeping this overview as detailed as possible to guarantee both the replicability of our annotations and the application of the proposed scheme to other genres not included in this work. The creation of a reliable corpus for cross-genre and diachronic study of CTs is a notable result, especially when considering the effort in the annotation of linguistic phenomena which lie at the semantic-pragmatic interface.

Although this newly annotated corpus does not solve all incompatibilities and differences surfacing at the theoretical level, it provides the community with a reference resource for the study of CTs in non-structured and semi-structured texts (e.g., news, travel reports, and travel guides) and their potential application in other NLP tasks.

### Genres and time

We adopt a broad perspective on texts and language assuming that robust NLP systems must work across genres (synchronic dimension) and time periods (diachronic dimension). This will facilitate their re-usability in different fields of study, and promote cross-fertilisation among disciplines.

We collected texts in English from three different genres: news, travel reports, and travel guides. For each genre, we gathered data published between the second half of the 1800s and the beginning of the 2000s. This variation in time and genres has allowed us to study the application of the proposed annotation scheme in different texts, testing its portability and genre-independence and, at the same time, it has made easier the emergence of genre specific aspects, showing consistency across time.

We selected the three aforementioned genres because we want to:

- systematically investigate the application of CTs to less structured texts, such as newspaper articles and travel reports;
- compare these documents with more structured ones, such as travel guides;
- investigate the relationships across these three genres that we see as a being part of a writing style continuum, with the newspaper articles and the travel guides as being at the two extreme poles, and the travel report as being a more fluid and hybrid category.

The collection of documents and its annotations compose the corpus, released to the public under the name of *Content Types Dataset version 1.5* (CTD v1.5).^4^

### Raw corpus statistics

The combination of the time and genre dimensions gives rise to six sub-corpora. For each of them, we obtained a collection of plain text documents, the *raw corpus*. As illustrated in Table 2, the raw corpus contains 279 documents, 183,517 tokens, and 20,190 clauses.

**Table 2:** Statistics on the raw corpus of *CTD v1.5*

Genre and time	Documents	Tokens	Clauses	Avg tok/doc	Avg tok/clause
Cont. news	84	32,086	3033	381.9	10.6
Hist. news	50	29,717	3821	594.3	7.8
Cont. travel reports	23	30,747	3969	1336.8	7.7
Hist. travel reports	25	31,690	3169	1267.6	10
Cont. guides	58	29,950	3102	516.4	9.6
Hist. guides	39	29,327	3096	751.9	9.5
Overall	279	183,517	20,190	657.8	9.1

In designing CTD v1.5, we wanted to keep a balanced combination for time and genre in terms of number of tokens and clauses. Furthermore, given the phenomenon under study, we decided to preserve documents’ integrity rather than truncating them. This last choice is reflected in the data with some sub-corpora (e.g., Historical News and Travel Reports) being slightly larger with respect to the others.

The Contemporary News sub-corpus has been created by selecting articles from available language resources, in particular the Wall Street Journal Corpus (Charniak et al., 2000) and the TempEval-3 dataset (UzZaman et al., 2013). This choice has the advantage of extending existing annotated data with additional layers and facilitating cross-fertilisation of tasks and data. Historical news were taken from Wikisource,^5^ a digital library of text transcriptions free of copyright. For both contemporary and historical news, we have restricted our selection by discarding editorials and commentary articles.

Historical travel reports and guides were extracted from a larger collection of travel writings downloaded from the Project Gutenberg website (Sprugnoli, 2018).^6^ Texts are chapters of books written by Anglo-American authors about Italy, published between 1860 and the 1920’s. In the historical period we considered, the distinction between reports and guides was not clear-cut as it is nowadays: reports of personal travel experiences were often mixed with practical recommendations and long disquisitions on art and history. Therefore, we adopt the distinction suggested by Santulli (2007b): travel narratives are those narrated in first person, while guidebooks are written in impersonal form.

Texts belonging to the Contemporary Travel Reports and Contemporary Travel Guides sub-corpora were taken from various sources on the Web. Guides are mainly from the WikiTravel portal^7^ and from the Lonely Planet website,^8^ to which we have asked for a non-profit use permit. In addition, we include one text belonging to the Manually Annotated Sub-Corpus (MASC) of American English (Ide et al., 2008). Contemporary travel reports are blog posts of several Anglo-American travellers: for each post we obtained the authorisation of re-use by the authors.

### Annotation study: model selection and adaptations

The development of the annotation model for CTs is based on a reconciliation of different theories and frameworks. The main principles of our annotation model are the following:

- the identification of the communicative function of a CT, i.e. its class, is determined by its content, i.e. the meaning, of the text segment in analysis;
- the identification of a CT class does not depend from other CTs;
- CTs make the communicative components of a discourse explicit.

The minimal textual unit which may express a CT is the clause, as it is also proposed by Cocco et al. (2011).^9^ Clauses are textual constituent units (Polanyi, 1988) defined as groups of words related to each other, containing a finite or non-finite predicate, while the subject may be implicit or shared with other clauses. This fine-grained level of the annotation has been selected as the outcome of an empirical analysis of the texts. Using sentences as minimal annotation units (as done in previous work, see Sect. 3) is acceptable if the writing style of the documents is well structured (e.g. movie reviews or scientific articles) and if the goal is to make explicit larger portions of a text, such as *argument zones*. Our analysis wants to avoid (over)simplifications and show the complexity of a sentence, and consequently, of a text. To further support our decision consider example (1). The sentence is composed by two clauses, separated by a “//”.

1. *I am writing on a fine terrace overlooking the sea,// where stone benches and tables are conveniently arranged for our use*. [Historical Travel Report: *13)SORRENTO, March 11th*]

According to our model, the two clauses in example (1) contain different CTs, the first is *narrating* what the main character is doing, whereas the second is *describing* the surrounding environment where the action takes place. A sentence-level (or even a paragraph-level) annotation would force the decision between one of the two interpretations, thus losing information, or impose a multi-label classification (Song et al., 2017).

The Content Type annotation scheme proposes seven classes. Five of them are shared by almost every theoretical framework presented in Sect. 2. Their specific naming follows Werlich’s typology since we found those labels more attuned with our task and transparent. The other two classes (**OTHER** and **NONE**) have been introduced to account for unclear and undefined cases. Below, we report the classes, their definitions, and examples. All examples have been extracted from CTD v1.5.

- **NARRATIVE**: a narrative CT contains an eventive or a stative predicate that can be anchored to a hypothetical or ideal timeline, even if not reported in a perfect sequential order, due, for example, to flashbacks:
  (2) *We left Cava on Wednesday, // and made the tour from there to Amalfi*. [Historical Travel Report: *13)SORRENTO, March 11th*]
    Clauses introducing a direct or a reported speech (in bold) are always to be annotated as **NARRATIVE**.
  (3) ***Mr Erdogan’s office said*** // *he had accepted the apology* [Contemporary News: *bbc_20130322_1353*]
    The factuality profile of the events does not influence the assignment of the category. Clauses with hypothetical, probable, uncertain, future events can be annotated as **NARRATIVE**.
  (4) *you can never be sure // whether they’ll be open // after snow has fallen*. [Contemporary Travel Report: *A stroll up Monte Terminillo*]
- **ARGUMENTATIVE**: an argumentative CT contains opinions and comments having explicit evaluations markers (in bold below):
  (5) *As for myself, I* ***hate*** *Viareggio at all seasons*. [Historical Travel Report: *6)Viareggio_February*]
  (6) *The American proposal is not an* ***adequate*** *basis for negotiation*. [Contemporary News: *wsj_0942.xml*]
- **DESCRIPTIVE**: a descriptive CT contains visible and/or invisible, tangible and/or intangible characteristics of entities, such as objects, persons, or locations. These characteristics have the goal of creating a mental picture of the entities in the reader’s mind;
  (7) *Some flags are lazily stirring over the entrance*. [Contemporary Travel Report: *Naples_by_Night*]
  (8) *She is a spirited creature , but with a fine balance of common sense*. [Historical Travel Report: *1)GENOA, February 19th*]
    They often include the presence of adjectives expressing size, colour, or shape of a person, a thing, an animal, or a place.
  (9) *His face had the normal amount of color in it* [Historical News: *Buffalo_Men_at_the_Execution*] Linguistic elements expressing spatial order are often present in case of descriptive CTs related to visible characteristics of entities:
  (10) *The road winds above, beneath, and beside rugged cliffs of great height*. [Historical Travel Report: *13)SORRENTO, March 11th*]
- **EXPOSITORY**: an expository CT contains generalisations with respect to a class. These clauses include linguistic expressions that make generic statements or refer to classes, or kinds, giving generic information about them. In the examples we mark in bold the linguistic expressions that trigger this type of CT.
  (11) *In Naples* ***every*** *pizzeria makes a decent pizza*. [Contemporary Guide: *Eat*]
  (12) ***The Roman*** *hates* ***the Piedmontese*** *and* ***the Neapolitan*** *and* ***the Bolognese***. [Historical Guide: *CHAPTER_II_Highways*]
- **INSTRUCTIVE**: an instructive CT expresses procedural information, such as the steps to be followed in a tour to reach a specific place, or orders;
  (13) *At last you cross that big road // and strike the limestone rock*. [Historical Travel Report: *6)Viareggio_February*]
  (14) *Flannel or silk should always be worn next to the skin*. [Historical Guide: GENERAL_HINTS]
- **NONE**: this class is reserved for clauses having mainly structural purposes, such as headers and titles.
  (15) *Jim Laurie, ABC News, Hong Kong*. [Contemporary News: *ABC1998 0108.1830.0711*]
  (16) *Day 271 Saturday 15th October 2016*. [Contemporary Travel Report: *Day_271_Naples*]
- **OTHER**: a CT of type **other** applies when none of the other classes can be identified. This includes clauses containing, for example, text in languages other than English, references to the reader, and citations of literary works:
  (17) *Buon appetito, ragazzi!* [Contemporary Guide: *Naples_on_a_plate_LP*]
  (18) *Your most interesting letter , Sir Philosopher , reached me at Gibraltar*. [Historical Travel Report: *1)GENOA, February 19th*]
  (19) *My soul to-day Is far away , Sailing the Vesuvian Bay; My winged boat, A bird afloat, Swims round the purple peaks remote *. [Historical Travel Report: *10)NAPLES, March 7th*]

For each of the aforementioned classes, a set of well-defined characteristics has been identified to avoid confusion. For instance, the presence of epistemic markers or markers of subjectivity (in bold in example (20)) is a distinguishing factor of the **ARGUMENTATIVE** class, preventing its confusion with the **DESCRIPTIVE** or the **NARRATIVE** ones:

(20) *This is another “Cornice Drive”*$_{DESCRIPTIVE}$ // *and* ***far finer*** [...] *than that along the Riviera.*$_{ARGUMENTATIVE}$ [Historical Travel Report: *13)SORRENTO, March 11th*]

Similarly, the fact that the interpretation of a clause is almost completely a-temporal, or lacks dynamism, are clear cues that the clause should be interpreted as a belonging to the **DESCRIPTIVE** class:

(21) *Over one of the doors is a Virgin and Child* $_{DESCRIPTIVE}$ [Historical Travel Report: *1)GENOA, February 19th*]

#### Annotation workflow

Three non-English native speakers participated in the annotation: all of them are expert linguists, two (A1 and A2) are also authors of this paper whereas the other (A3) learned the task by independently reading the annotation guidelines. The annotation was carried out following a multi-step process and using the web-based tool CAT (Bartalesi Lenzi et al., 2012). In the first phase, annotators A1 and A2 were allowed to discuss disagreements based on a trial corpus suggesting revisions to improve the first version of the guidelines. The trial corpus contains a random sample of 16 documents (4 Contemporary Guides, 4 Historical Guides, 2 Historical Travel Reports, and 6 Contemporary News) that compose CTD v1.5. In the second phase, inter-annotator agreement between A1 and A3 was calculated on a subset of CTD v1.5 (see Sect. 4.3.2). In the final phase, after the computation of the Inter-Annotator Agreement (IAA), the rest of the dataset was annotated by applying the latest version of the guidelines which includes detailed descriptions of the classes, examples from each sub-corpora, and preference constraints to discriminate ambiguous cases. The final version of the corpus has been annotated independently and with an equal distribution of the documents and workload by A1 and A3.

#### Inter-annotator agreement

The inter-annotator agreement is calculated on a sub-set of 19,300 tokens of the corpus, balanced for genre and time, representing $\approx$10% of the tokens of each sub-corpora.^10^ Table 4 reports the Cohen’s kappa micro-average on the number of tokens per clause for each sub-corpus.

**Table 3:** Statistics of the subset annotated for the IAA

CT	Cont guides	Hist guides	Cont news	Hist news	Cont reports	Hist reports	IAA subset
NARR.	17.8	24.4	73.1	65.7	55.1	39.3	49.5
DESC.	34.7	47.6	6.6	3.9	10.9	15.3	16.5
ARGU.	10.9	17.1	19.8	23.9	15.1	30.4	20.5
INST.	19.2	5.7	–	–	5.4	0.9	4.1
EXPO.	7.8	0.4	–	2.4	6.2	1.5	3.0
OTHER	0.9	2.4	–	–	3.5	12	3.1
NONE	8.7	2.4	0.5	4.1	3.8	0.6	3.3

Figures report distribution of the classes in percentage

**Table 4:** Inter-annotator agreement: Cohen’s kappa micro-average calculated at token level

CT	Cont guide	Hist guide	Cont news	Hist news	Cont report	Hist report	Average per class
NARR.	0.97	0.79	0.86	0.89	0.84	0.88	0.87
DESC.	0.82	0.89	0.84	0.76	0.75	0.86	0.82
ARGU.	0.80	0.85	0.82	0.94	0.9	0.9	0.87
INST.	0.97	0.90	–	–	0.86	0.65	0.84
EXPO.	0.79	0.89	–	0.81	1	0.93	0.88
OTHER	1	1	–	–	0.95	0.92	0.97
NONE	1	1	1	1	1	1	1
Overall avg.	0.91	0.90	0.88	0.88	0.9	0.87	0.89

All classes have high average scores, reaching or exceeding .80, a value usually sets as a threshold that guarantees good annotation quality (Artstein & Poesio, 2008). However, variations can be spotted in the sub-corpora. For example, the agreement on **INSTRUCTIVE** CTs is as low as .65 on Historical Reports, but this class has also been annotated only twice whereas in the other sub-corpora, e.g. Contemporary Guides and Historical Guides, it appears 14 and 41 times, respectively, and it has higher agreement scores.

By analysing the annotations, we noticed that one of the main sources of disagreement was the identification of clause boundaries, in particular in case of *to*-infinitive and parenthetical clauses.

(22) Annotator 1: *The Spanish Bourbons were the last // to rule in Naples* Annotator 2: *The Spanish Bourbons were the last to rule in Naples* [Historical Travel Report: *NAPLES_CATHEDRAL_CITIES_1*]
(23) Annotator 1: *Weisfield’s, based in Seattle, Wash., currently operates 87 specialty jewelry stores in nine states*
  Annotator 2: *Weisfield’s,// based in Seattle, Wash.,// currently operates 87 specialty jewelry stores in nine states* [Contemporary News: *wsj_0505*]

In addition, we observed some confusion between **EXPOSITORY** and **DESCRIP TIVE** CTs: this can be due to the fact that both classes lack dynamism and tend to be expressed using a similar tense (e.g. present tense), differentiating only for the genericity of their referents.

(24) Annotator 1: *[The paintings usually depict a serene and beautiful landscape, such as in Giacinto Gigante’s Panorama of Naples Viewed from the Conocchia]_*$_{EXPOSITORY}$
  Annotator 2: *The paintings usually depict a serene and beautiful landscape, such as in Giacinto Gigante’s Panorama of Naples Viewed from the Conocchia]_*$_{DESCRIPTIVE}$ [Contemporary Guide: *CERTOSA_DI_SAN_MARTINO _DK*]

Another issue concerns the annotation of **ARGUMENTATIVE** CTs. Besides the good agreement, the presence of even a single polarised words, such an evaluative adjective, led one of the annotators to prefer that class with respect to the others.

(25) Annotator 1: *[Augustus did much for Neapolis,]_*$_{NARRATIVE}$ // [and Tiberius sought refuge in that entrancing island, Capri,]_$_{NARRATIVE}$ // *[where to this day his* ***infamies*** *are a byword*]_$_{ARGUMENTATIVE}$
  *Annotator 2:* *[Augustus did much for Neapolis,]*_$_{NARRATIVE}$ // [*and Tiberius sought refuge in that entrancing island, Capri,*]_$_{NARRATIVE}$ // [*where to this day his infamies are a byword*]_$_{NARRATIVE}$ [*Historical Guide:* *NAPLES_CATHEDRAL_CITIES_1*]

We also calculated the IAA at clause level. We performed a best-effort alignment of the clauses considering valid those with an overlap of at least 60% of the tokens. This results in the exclusion of 228 clauses (i.e., 11.88% of the clauses in the subset of files annotated for the IAA in the Gold Standard), reaching a Cohen’s kappa of 0.91. When including the mismatched clauses as errors^11^ in the IAA score, and thus considering a stricter evaluation setting, the kappa drops to 0.61.

#### Final corpus

Table 5 illustrates the composition of CTD v1.5 reporting the distribution (in percentages) of the seven classes in all sub-corpora. Disagreements registered in the IAA were reconciled and the resulting annotations has been integrated in the final corpus. **NARRATIVE** clauses cover more than 50% of the annotated CTs but their presence differs across the sub-corpora. In particular, they correspond to the strong majority (> 70%) in both Contemporary and Historical News. They are the most frequent class also in Reports, whereas Guides present a more balanced distribution. **DESCRIPTIVE** CTs characterise Contemporary and Historical Guides and, in general, are more present in the travel domain given that they tend to provide an overview of places that can be experienced during a journey (they are above 15% in Reports). **ARGUMENTATIVE** CTs are above 10% in all sub-corpora: they are particularly relevant in Reports, which contain opinions and personal feelings about places and people met by the author during his/her journey. **INSTRUCTIVE** and **EXPOSITORY** CTs have a very low frequency (< 3%) in News and Reports but, on the contrary, characterise Guides. This confirms previous studies that indicate procedural text as a distinctive element of tourist guides (Santulli, 2007a), assigning to guidebooks a central role in the creation, maintenance and use of generalisations and stereotypes about places and populations (Bender et al., 2013). The highest percentage of **OTHER** CTs is found in Historical Reports where there are several occurrences of literary citations and various cases of clauses in languages other than English, related to the phenomenon of code-mixing (Sprugnoli et al., 2017b). Moreover, some of the texts in this latter sub-corpus are written as letters, thus they contain direct references to the reader. As for the **NONE** CTs, they are particularly frequent in Contemporary Guides: those texts are quite structured, divided in different subsections, each having the title annotated as **NONE**.

**Table 5:** Statistics of the annotated data

CT	Cont guides	Hist guides	Cont news	Hist news	Cont reports	Hist reports	CTD v1.5
NARR.	14.4	31.4	76.3	72.5	59.5	54.9	52.5
DESC.	36.9	29.5	6.5	6.8	17.4	15.0	18.2
ARGU.	10.8	14.8	14.0	12.8	15.5	19.8	14.6
INST.	23.7	11.3	0.2	0.7	1.9	0.2	5.9
EXPO.	4.7	8.5	1.9	0.6	1.8	2.7	3.2
OTHER	1.3	2.4	0.5	1.2	2.2	6.1	2.3
NONE	8.2	1.9	0.5	5.4	1.6	1.2	3.2

Figures report distribution of the classes in percentages in each sub-corpus and in CTD v1.5

When aggregating the data per genre, we observe that the distribution of CTs is statistically significant (*p* < 0.01, calculated with the Z test) across genres, with the sole exception of the **ARGUMENTATIVE** CTs between News and Guides. As for the temporal dimension (Historical vs. Contemporary) the distribution of CTs is statistically significant for all classes (*p* < 0.01, calculated with the Z test) except for **NONE**. The distribution of the annotations across the sub-corpora supports our intuition about the emergence of specific properties of text types from the distribution of CTs. The three text types in our corpus distinguish from each other for the presence of one or more prevalent CTs that tend to dominate on the others. For instance, news and travel reports focus more on the narration of “things that happened”, while guides tends to describe situations. However, travel report differentiates from the other two because of an almost equal distribution of the DESCRIPTIVE and ARGUMENTATIVE classes. Similarly, guides differ from the other two text types because of a more varied presence of the other classes, namely DESCRIPTIVE and INSTRUCTIVE. By using CTs and their distribution, it appears more clearly that texts are complex entities and that their classification in “ideal” types may result in oversimplifications. In a diachronic perspective, the variation in the distributions of the CTs between the historical and contemporary texts is an additional empirical evidence of the changes in the writing styles and in the text types. The most radical changes can be seen in the guides, with a reduction of the presence of NARRATIVE, EXPOSITORY, and ARGUMENTATIVE classes, cues of personal experience, and increase of less subjective ones such as DESCRIPTIVE and INSTRUCTIVE. Less radical changes can also be observed in the news and travel report texts.

## Experiments

We have conducted a series of experiments on the automatic identification of CTs. We evaluate on three different scenarios against a fixed test set. First, we report results of different models’ architectures. Second, we deal with the impact of clause’s extent prediction (*silver data*) on the labelling of CTs, to assess the application of the model to other data sets. Third, to evaluate the impact of time and genre, we train separate models for each of these dimensions and test them against in- and across-domain data (*across genre* and *across time* evaluation).

*Data Representation and Evaluation* CTD v1.5 has been split in training (70%), development (10%), and test (20%) according to the distribution of CTs. The dataset is unbalanced with four classes representing almost a quarter of the data (overall the classes INSTRUCTIVE, EXPOSITORY, OTHER, and NONE represents 23.77% of the occurrences).

We have approached the identification of CTs as a sequence labelling problem rather than a text classification task, training the classifier on texts split by clauses instead of sentences. We use the BIO (Begin-Inside-Outside) format so that each token in a clause gets one of following 14 labels: B-NARRATIVE, I-NARRATIVE, B-DESCRIPTIVE, I-DESCRIPTIVE, B-ARGUMENTATIVE, I-ARGUMENTATIVE, B-INSTRUCTIVE, I-INSTRUCTIVE, B-EXPOSITO-RY, I-EXPOSITORY, B-OTHER, I-OTHER, B-NONE, I-NONE.

In the following subsections, we report overall Precision, Recall and F1 scores and focus on detailed results per each of the seven CT classes only for the best model(s). Evaluations are performed at token level, and for each token, the predicted label must match exactly the gold data label. This will avoid the introduction of a further evaluation aspect, namely (implicit) clause boundary detection by means of CTs. However, the impact of clause boundary detection is taken into account in our second evaluation scenario (see Sect. 5.3).

### Contribution of contextualised embeddings

The first set of experiments investigates the impact of contextualised word embeddings, namely ELMo (Peters et al., 2018), for this task.

We used a common architecture based on a bi-directional Long Short-Term Memory (bi-LSTM) network. We used a publicly available^12^ and state-of-the-art implementation (Reimers & Gurevych, 2017b). LSTM-networks have been successfully used in many Natural Language Processing (NLP) tasks and have achieved state-of-the-art results for many sequence labelling tasks (Chiu & Nichols, 2016; Kiperwasser & Goldberg, 2016; Ma & Hovy, 2016; Malca & Reichart, 2018; Søgaard & Goldberg, 2016).

Each clause, in this case, is represented as a sequence of tokens. Each token in a clause is mapped to a pre-trained word embeddings. In addition to this, we also used 30-dimensional character embedding representations using a Convolutional Neural Network (CNN) (Ma & Hovy, 2016). The word and character embeddings are concatenated and used for the bi-LSTM encoder. Two bi-LSTM layers (with 100 recurrent units each) are used. The output of the bi-LSTM layers is then passed to a CRF classifier to produce the most likely tag sequence at token level. The network has been trained using **adam** optimizer (Kingma & Ba, 2014) and a variational dropout.^13^

**Fig. 1:**
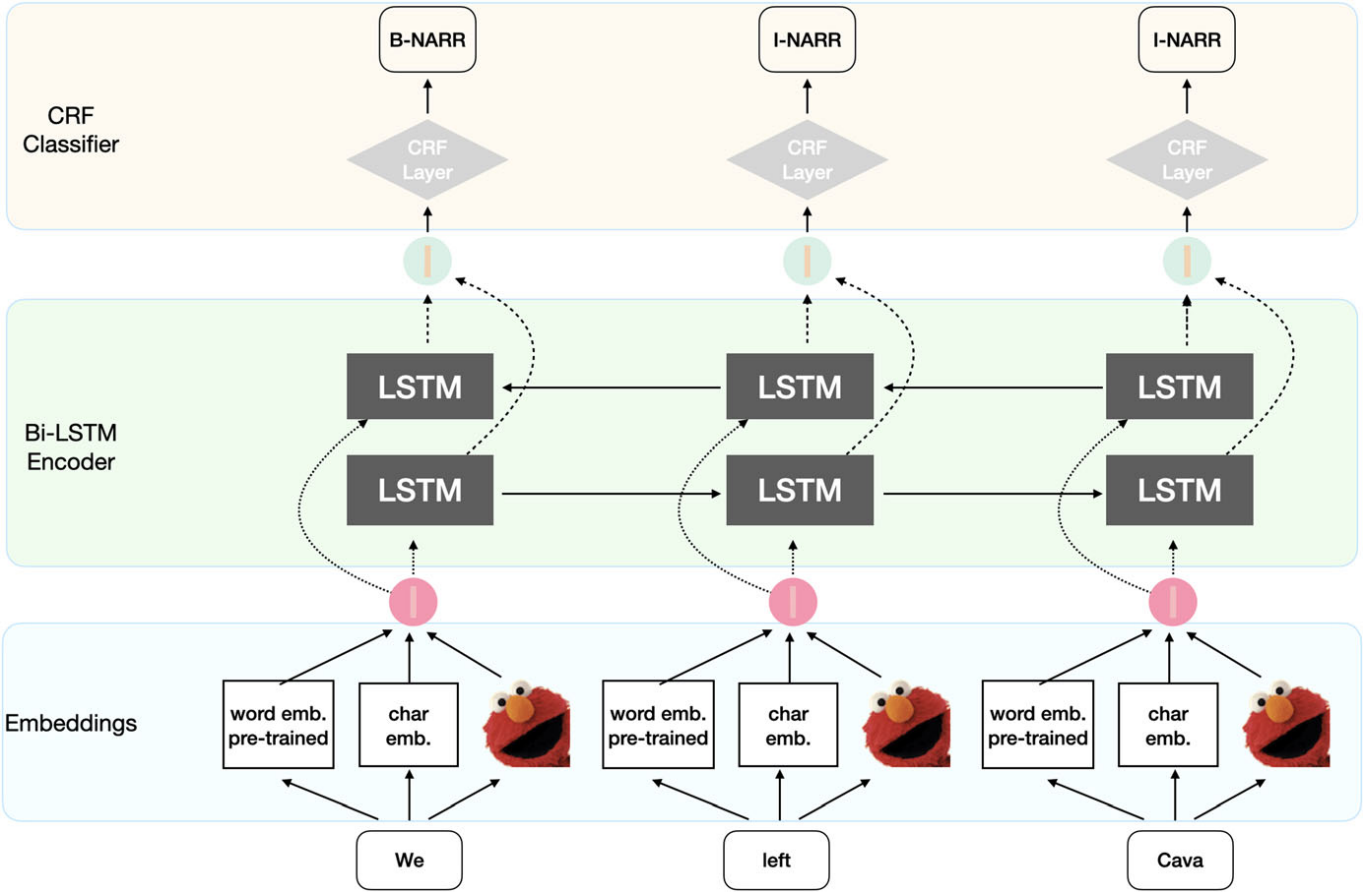
Architecture of the **bi-LSTM-CRF ELMo+** network with CRF classifier. **bi-LSTM-CRF standard** and **bi-LSTM-CRF ELMo** are variations of this architecture

We differentiate the bi-LSTM architectures only with respect to the word embedding representations used for the network initialisation. In the first version, **bi-LSTM-CRF standard**, we use 300-dimensional pre-trained word embeddings, namely Komninos and Manandhar (2016), who implement a skip-gram model using structural information from dependency graphs to train the embedding model. The second version, called **bi-LSTM-CRF ELMo**, uses fine-tuned ELMo embeddings, and, finally, the third version, **bi-LSTM-CRF ELMo+** concatenates the Komninos and Manandhar (2016) embeddings with fine-tuned ELMo. When using ELMo, we followed Reimers and Gurevych (2019) by taking the weighted average of only the first two layers of the ELMo embeddings as it has shown to provide the best results on multiple sequence labelling tasks. Figure 1 graphically illustrates the full architecture of the bi-LSTM-CRF network.

Results of the models are reported in Table 6. We implemented two baselines. The first is a most frequent class baseline. In this case, we always assign the most frequent CT (i.e. **NARRATIVE**). The second baseline is a feature-based Conditional Random Field (CRF) model (Lafferty et al., 2001).^14^ We selected surface features based on lemmas, POS, morphological features, and dependency features using a context window of two. Features have been extracted with UDPipe 2.6 via LINDAT UDPipe REST Service^15^ using the English **partut-ud-2.6** model. The CRF model has been implemented using the CRF++ toolkit^16^ with default parameters.

The low performance of the CRF model indicates that surface features (i.e., morpho-syntax) are unable to correctly grasp the semantics of the clauses necessary to identify the CT labels. On the other hand, all versions of the bi-LSTM network obtain very good results outperforming both baselines. Both versions with ELMo have better results than the network using pre-trained embeddings alone, reaching an maximum average F1 score of 73.34% when concatenated with the pre-trained embeddings. The use of ELMo is beneficial as the network can rely on two different input representations should some relevant information to solve the task not being present in one or the other embedding representation.

**Table 6:** Results on the test set

Model	P	R	F1
**Majority class baseline**	52.05	52.04	52.04
**CRF baseline**	11.15	31.70	16.49
**bi-LSTM-CRF standard**	70.74$_{0.15}$	70.32$_{0.13}$	70.56$_{0.14}$
**bi-LSTM-CRF ELMo**	71.92$_{0.80}$	73.18$_{0.78}$	72.55$_{0.78}$
**bi-LSTM-CRF ELMo+**	**73.36**$_{0.56}$	**73.32**$_{0.55}$	**73.34**$_{0.59}$

Scores for bi-LSTM models are based on the average of P, R, and F1 per class over five multiple runs. Subscript numbers indicates standard deviation. **Bold** numbers denote the best results

The concatenation of pre-trained embeddings with ELMo, however, is a variable that may impact on the results (Reimers & Gurevych, 2017a). We further evaluated the ELMo-based bi-LSTM architecture with three additional pre-trained word embeddings that use different approaches to generate their representations, namely:

- **Word2Vec** (Mikolov et al., 2013): we used the pre-trained Google News corpus (3 billion tokens) skip-gram word vector model with 300 dimensions;
- **GloVe** (Pennington et al., 2014): we used the 300 dimensional pre-trained vectors obtained from Wikipedia and Gigaword corpus (6 billion tokens);
- **FastText** (Bojanowski et al., 2017): we used the 300 dimensional pre-trained vectors obtained from the Common Crawl project (600 billion words).

The results, reported in Table 7, show that varying the concatenated pre-trained embedding changes the final results. Besides being minimal, all other embeddings representations perform better than the Komninos and Manandhar (2016) with **Word2Vec** obtaining the best results. We thus decide to keep experimenting using the **bi-LSTM-CRF ELMo+** network with **Word2Vec** embeddings.

**Table 7:** Results on the test set obtained with different pre-trained word vectors

Embedding model	P	R	F1
Komninos and Manandhar (2016)	73.36$_{0.56}$	73.32$_{0.55}$	73.34$_{0.59}$
** Word2Vec**	**73.86**$_{0.95}$	**73.80**$_{0.96}$	**73.82**$_{0.94}$
** GloVe**	73.58$_{0.58}$	73.48$_{0.62}$	73.53$_{0.59}$
** FastText**	73.58$_{0.79}$	73.46$_{0.77}$	73.51$_{0.78}$

Scores are the average of P, R, and F1 per class over five multiple runs. Numbers subscripts indicates standard deviation

### Discussion on the best model

Table 8 reports the scores per CT of the best model, i.e. **bi-LSTM-CRF ELMo+** with **word2vec** embeddings, reaching an overall 74.99 F1 score. The model can generalise quite well across CTs, although the distribution of the data in the training set has an impact. For instance, the model obtains the best results on **NARRATIVE**, which is also the most frequent CT in CTD v1.5. However, it scores high also for less frequent CTs, such as **NONE** and **INSTRUCTIVE**. Some of the hardest cases are represented by the **EXPOSITORY** and **OTHER** CTs. **DESCRIPTIVE** and **ARGUMENTATIVE** CTs, which together represent  33% of the classes, obtain satisfying results although divergent. In particular, we observe a good performance of the **DESCRIPTIVE** CT (68.73%) and only satisfying results for the **ARGUMENTATIVE** ones (59.81%) indicating a higher level of complexity in identifying this class as suggested by the low Recall (only 55.75%).

**Table 8:** Best model—overall results and per CT class

CT class	P	R	F1
** NARRATIVE**	74.72	85.96	79.90
** DESCRIPTIVE**	69.77	67.73	68.73
** ARGUMENTATIVE**	64.51	55.75	59.81
** INSTRUCTIVE**	68.07	52.31	59.16
** EXPOSITORY**	62.86	14.67	23.78
** OTHER**	51.92	36.49	42.86
** NONE**	86.02	93.02	89.39
** Overall**	75.04	74.95	74.99

**Fig. 2:**
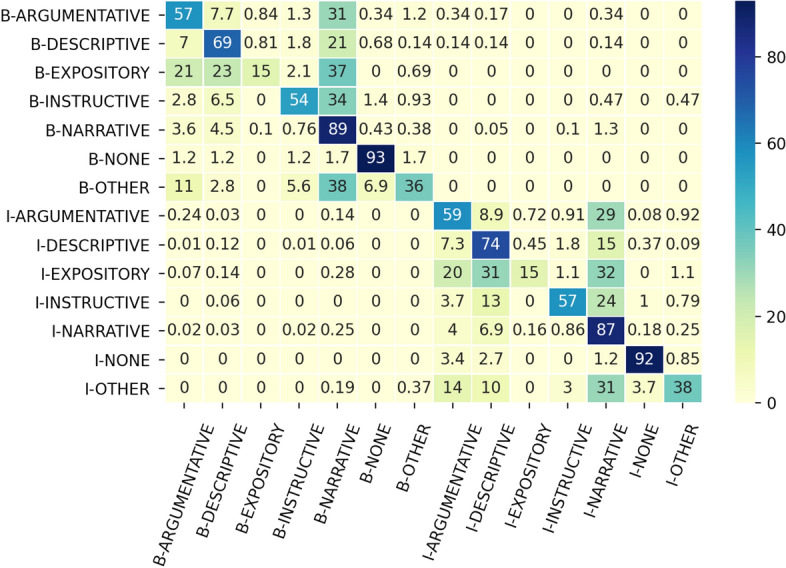
Normalised confusion matrix from the best performing model per CT class label at token level. Row labels are the gold labels, column labels are predictions. Values are percentages. The sum of the values in each row is 100%

*Error Analysis* The error analysis based on the normalised confusion matrix in Fig. 2 shows additional details. Errors across *B*-labels and *I*-labels are very limited: only in one they are higher than 1% ( *B-NARRATIVE* as *I-NARRATIVE*). This reflects errors in detecting discontinuous CTs that involve parenthetical or non-restrictive relative clauses.

(26) Gold: *Doctor Tyree once insisted McManus take the sick man into the police office there , // but McManus refused , // saying*$_{B-NARRATIVE}$ // *more*$_{B-NARRATIVE}$ *persons would be exposed .* System: *Doctor Tyree once insisted McManus take the sick man into the police office there , // but McManus refused , // saying*$_{B-NARRATIVE}$ *more*$_{I-NARRATIVE}$ *persons would be exposed .*

On the basis of the distribution of the labels in the training set, it is not surprising that the majority class, i.e. **NARRATIVE**, tends to be assigned more often, representing almost a quarter of the errors across all the classes. We interpret this as a cue of the sensitivity of the model to the training data where, in absence of any better cue, it assigns the most frequent label. In the following paragraphs we analyse in details the misclassification on classes other than **NARRATIVE**.

**EXPOSITORY** is often confused with **ARGUMENTATIVE** and **DESCRIPTIVE**. This shows that distinguishing between the expression of an opinion, or a description of a place or of a person, and the expression of a generalisation with respect to a class is quite difficult, as it requires fine-grained linguistic analysis to interpret the differences. This type of errors was also spotted as a case of disagreement between the annotators, indicating that such distinctions are not easy to identify. We can also observe how the misclassification is consistent between the B-* (20%) and I-* (21%) tags.

(27) Clause: *and nearly every one writes impressions and descriptions of the performance.* [*EXPOSITORY*$_{GOLD}$-*ARGUMENTATIVE*$_{SYS}$]
(28) Clause: *Flowers are one of the economies of San Remo.* [*EXPOSITORY*$_{GOLD}$-*DESCRIPTIVE*$_{SYS}$]

A further frequent mismatch concerns **ARGUMENTATIVE** and **DESCRIPTIVE**. More than 7% of the errors in the labels of these classes is systematically confused, indicating that there is some overlap in the way these classes are realised, although their distinction is pretty neat.

(29) Clause: *which are richly carved and of a stone warm and creamy in tone,* [*DESCRIPTIVE*$_{GOLD}$-*ARGUMENTATIVE*$_{SYS}$]

An additional relevant error is between **INSTRUCTIVE** and **DESCRIPTIVE**. Once again, the system is not able to capture subtle differences in the realisation of this class while relying too much on surface realisations of the clause.

(30) Clause: *Numerous other walks and excursions will easily be discovered by inquiry.* [*INSTRUCTIVE*$_{GOLD}$-*DESCRIPTIVE*$_{SYS}$]

The class **OTHER** tends to be confused most of the time. As a matter of fact, this class is a sort of bucket where pretty diverse clauses can end up because none of the previous labels could reliably be applied. Nevertheless, humans do not seem to have much problems in identifying it, reaching a very high agreement in the different sub-corpora (Cohen’s kappa ranges between .92 for Historical Reports to 1.0 for Historical and Contemporary Guides).

Finally, **NARRATIVE** is not immune to misclassifications: most of the errors tend to end up either in the **ARGUMENTATIVE** (3.6% *B-ARGUMENTATIVE* and 4% *I-ARGUMENTATIVE*) and in the **DESCRIPTIVE** (4.5% *B-DESCRIPTIVE* and 6.9% *I-DESCRIPTIVE*) classes. This is not surprising given that the definition of the **NARRATIVE** class includes stative predicates thus confusing the model.

(31) Clause: *for the greater pleasure of wandering at will through the charming, picturesque cloisters,* [*NARRATIVE*$_{GOLD}$-*ARGUMENTATIVE*$_{SYS}$]
(32) Clause: *Standing upon the Belvedere of San Martino,* [*NARRATIVE*$_{GOLD}$-*DESCRIPTIVE*$_{SYS}$]

*Clause-based Evaluation* To gain a more comprehensive overview of the performance of the system, we have conducted an additional evaluation at clause level. In this setting, we have considered only the class label for the entire clause. Table 9 illustrates the results of the best system and compare it against the two baselines, i.e., most frequent and CRF.

**Table 9:** Results for clause-based evaluation. Scores for bi-LSTM model refers to the best model only

Model	P	R	F1
** Majority class baseline**	52.02	52.02	52.02
** CRF baseline**	60.00	60.06	60.03
** bi-LSTM-CRF ELMo+**	**75.36**	**75.73**	**75.77**

**Bold** numbers denote the best results

This evaluation scenario is more lenient. Although the majority baseline obtains the same results, we observe big improvement of the CRF baseline (+43.54 points in terms of F1 score, see Table 6). The result is somehow misleading since the distribution of the errors across the classes is the same that we obtain for the token-based evaluation. The improved results are only a distortion due to the way in which the clause-level evaluation is computed. In comparison, the increase in performance of the bi-LSTM model is modest, only ~ 2 points in F1. Such a limited increase when compared to the token-based evaluation indicates, on the other hand, a major robustness and reliability of the best model.

### Impact of silver data on clause boundary detection

One critical aspect that affects this task, especially in an end-to-end scenario (i.e. from raw text to full predictions), is clause boundary. The results of the experiments described so far use manually determined (i.e. gold) clause boundaries.

In these experiments we compared two different approaches of automatically segmenting sentences in clauses. The first approach uses a dedicated system, SPADE (Soricut & Marcu, 2003). SPADE is a rule-based probabilistic model of discourse parsing that uses syntactic and lexical features to generate basic discourse segments units (DSUs) corresponding to clauses or clause-like units. In this scenario, we have first applied SPADE to the test data, and then run our best model. The second approach exploits the token-based representation of CTs. In particular, we reconstructed the original sentences of each document but maintained the BIO representations of CTs. We then trained a new model using sentences as input and applied it to the test data.^17^

As our aim is to evaluate the impact of automatically segmented documents in clauses against manual segmentation, we evaluated the performance of the trained models for CT detection by taking into account both the segmentation in clauses and the predicted CT labels. In this case, each token was considered as correctly classified if there is a perfect match both for the predicted CT class and if the token belongs to the same clause as the gold standard. Table 10 illustrates the results clearly showing that the use of automatically generated, i.e. silver, clause segments has a negative impact on the performances of the model. In particular, the F1 score is slightly above 40% when using SPADE, while it drops below 60% when using the full sentence input.

**Table 10:** Results on Gold and Silver clause segmentation

Clause segmentation	P	R	F1
**Gold clause**	75.04	74.95	74.99
**SPADE—clause span and label**	41.97	44.40	43.15
**Full sentence—clause span and label**	61.28	60.24	60.76

All results are for the best models only

Clearly this is a more challenging evaluation setting since the system has to predict both class labels and clause boundaries. However, we deem this evaluation scenario useful to provide a lower bound of the performances of the model, and especially, of the impact of the correct clause segmentation on CT classification. The use of automatically generated clause segments with SPADE has a negative impact, with the scores dropping below 50%. Segmentation mismatches are the primary source of errors and have the biggest impact in downgrading the system’s performance. We conducted an exploratory study to check the quality of SPADE segmentation against the manual data by selecting a random sample of articles from the training set containing a total of 3603 clauses manually annotated. We applied SPADE and then evaluated how many times the manual and the automatic segmentation match. We observed that only in 51.87% of cases there was a perfect match with the manually annotated data. In the remainder of the cases, there was a partial match, where at most two gold clauses were segmented as one by SPADE. In no case, SPADE generated clause segments that have no match with the gold data. We also observed that SPADE tends to generate more segments than the manual data (3920 segments automatically generated *vs*. 3603 manual segments, respectively).

To confirm our analysis of the errors, in a further evaluation setting that excludes the clause segment constraint, the F1 score for SPADE jumps to 71.27%, while the full sentence input is almost on a par with the best model (74.19% sentence input *vs.* 74.99% for the best model). For the full sentence method, the majority of the errors are due to mismatches of B-* and I-* tags rather then the class label (e.g, NARRATIVE *vs.* ARGUMENTATIVE).

### Across genre and across time periods experiments

In the last set of experiments we investigated the impact of the two major dimensions that have been used in the creation of CTD v1.5, namely *time* and *genre*. In Sect. 4, we have illustrated that distribution of CTs is almost always statistically significant across genres and time. This makes interesting to investigate if models trained on different genres (*across genre evaluation*) or using documents published at different moments in time (*across time evaluation*), have different performances when tested both on their corresponding test sets and across them. We have designed these experiments as follows: first, we have aggregated the CTD v1.5 documents per genre, and subsequently, per time. This results in five different sub-corpora: three based on genres only (News, Guides, and Reports), and two based on time (Contemporary and Historical).

For each combination of train and test data (i.e. in-domain and across-domain), we re-trained our model (**bi-LSTM-CRF ELMo+)** and evaluated it against all test distributions, i.e., same sub-corpus and across sub-corpora. Results for the experiments on genres are illustrated in Table 11, while those for time periods in Table 12.

**Table 11:** Cross train and test across genres

Test $\rightarrow$	News			Guides			Reports		
Train $\downarrow$	P	R	F1	P	R	F1	P	R	F1
News	73.37$_{1.73}$	74.45$_{1.26}$	73.91$_{1.46}$	38.70$_{2.03}$	39.84$_{2.93}$	39.24$_{2.41}$	57.68$_{1.35}$	61.20$_{0.68}$	59.36$_{0.96}$
Guides	63.42$_{1.90}$	64.38$_{2.04}$	63.90$_{1.95}$	64.82$_{0.89}$	65.84$_{0.75}$	65.32$_{0.81}$	53.70$_{2.46}$	55.32$_{2.74}$	54.50$_{2.58}$
Reports	75.37$_{1.25}$	77.18$_{1.08}$	76.27$_{1.07}$	50.18$_{0.36}$	50.80$_{0.50}$	50.47$_{0.42}$	68.12$_{0.44}$	70.00$_{0.60}$	69.04$_{0.50}$

Scores are the average of P, R and F1 score per class over five multiple runs. Numbers subscripts indicates standard deviation

**Table 12:** Cross Train and Test across Time Periods

Test $\rightarrow$	Contemporary			Historical		
Train $\downarrow$	P	R	F1	P	R	F1
Contemporary	72.00$_{0.86}$	72.98$_{0.83}$	72.49$_{0.83}$	66.68$_{0.97}$	68.42$_{1.29}$	67.53$_{1.13}$
Historical	66.56$_{1.19}$	67.40$_{1.30}$	66.95$_{1.24}$	71.65$_{1.19}$	73.23$_{0.67}$	72.42$_{0.86}$

Scores are the average of P, R and F1 score per class over five multiple runs. Numbers subscripts indicates standard deviation

**Fig. 3:**
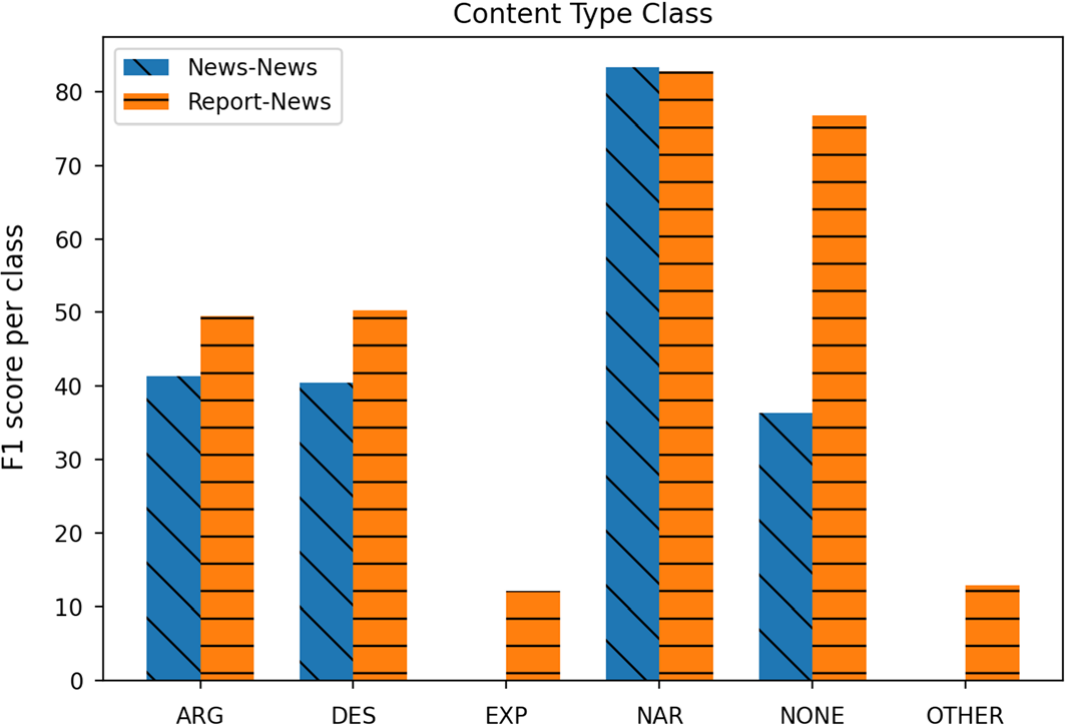
CT prediction per class on the News test set: in blue with oblique lines the results of the same data distribution model (i.e. train on News) and in orange with horizontal lines those for the cross-data distribution model (i.e. train on Report)

Not surprisingly, in both experiment settings, models trained and applied on the same data distributions obtain better results than when applied across them. This is in line with results from previous work in domain adaptation (Plank & Van Noord, 2011; Ruder & Plank, 2017). However, in the across genre evaluation setting, the model trained on the Reports distribution obtains better results in the News test than the model trained on the same data distribution, i.e. News training. As this is quite a peculiar behaviour, we ran the respective best performing models, the one trained on the News and that trained on Reports, against the News test set, to further analyse their performance with respect to each CT. The results in Fig. 3 show that the model trained on the Report data (orange column with horizontal line) outperforms the model trained on the News data (blue column with diagonal line) in five out of the six available CTs (namely, **ARGUMENTATIVE**, **DESCRIPTIVE**, **EXPOSITORY**, **NONE**, **OTHER**) and obtains very competitive results for **NARRATIVE**. As it already appears from Table 5, the distributions of CTs in these two genres is not balanced and this could be considered a factor explaining this different behaviour. Such imbalance of the distribution of CTs is mirrored in the respective training sets: the News training has only 7.07% of all clauses labeled as **DESCRIPTIVE**, 15.00% as **ARGUMENTATIVE**, less than 1% each for **NONE** and **OTHER**, and the large majority (74.96%) being **NARRATIVE**. On the other hand, the Report training seems more balanced. Although the **NARRATIVE** CT still remains the most frequent (58.92%), **DESCRIPTIVE** and **ARGUMENTATIVE** CTs (16.50% and 16.70%, respectively) have almost the same proportion, and **NONE** and **OTHER** are more frequent. Besides differences in the writing style between news and travel reports, the different CTs’ distribution makes the Reports model more robust, especially on those cases where the News training is weaker.

The cross-test results on the Guides data further highlight the differences among the three genres. Although the results are not directly comparable, it clearly appears that the identification of CTs is sensitive to genres and their properties. This confirms our initial intuitions about the differences among the three genres and further support the observations based on the annotated data. News and Guides position at the opposite of a imaginary continuum of difficulty, with the former being the easiest and the latter being the hardest, with Reports appearing somewhere in the middle.

When focusing on the across time period evaluation, we observe that in both corresponding test distributions, the trained models obtain good performances, well above 70%. The losses in the cross-test settings are limited, with the lowest for the model trained on Contemporary data (~ 5 points lower both for Precision and Recall). When compared to the across genre evaluation setting, it seems that time has a lower impact on the performances of the models, both in- and across data distributions, suggesting that changes in the CT structure of the texts seem minimal.

## Conclusion and future work

On the basis of the work presented in the previous sections, we summarise the answers to our three research questions posed in the Introduction (Sect. 1) as follows.

First, we have proposed a new empirical framework introducing the notion of content types that builds upon Werlich (1976)’s categories and attempts at reconciling existing differences across theoretical frameworks. This has been formalised in new annotation guidelines, and a large annotated corpus that, for the first time, allows a systematic study of CTs across texts belonging to different genres and published at different moments in time.

Second, we have successfully applied neural networks (i.e., bi-LSTM) to address the automatic classification of CTs. More specifically, we have shown the impact of different word embeddings in the task, comparing the use of non-contextualised *vs*. contextualised representations providing additional evidence in support of the use of contextualised embeddings. We also tested the value of concatenating different pre-trained word embeddings with the contextualised ones. In the perspective of developing an end-to-end system, we have evaluated two approaches for the automatic segmentation of sentences into clauses. Results of these experiments provide a lower bound of the performances of the model.

Third, we successfully experimented the portability of the annotations across genres and time periods. In this case, we observe a higher sensitivity of the trained models to text genres with respect to time, confirming our corpus observations on the distribution of CTs in different genres.

The genre and temporal diversity of our dataset is not ample and this constitutes a limitation for our study. Future works should focus in overcoming this aspect by extending the current CTD corpus with texts from other genres, domains, and time periods so to further improve the portability of the trained model and gain better data-driven insights on across genre and across time textual properties. In particular, it would be interesting to add non-narrative texts, such as manuals, school textbooks, and legal documents. More experiments can also be envisaged using other architectures and other types of embeddings, for example BERT (Devlin et al., 2018) that proved to have a strong impact in state-of-the-art NLP tasks. Finally, the annotation scheme could be additionally enriched with a set of special attributes that signal whether a CT is part or not of a direct or reported speech. This difference is useful as it may have an impact on other applications, such as the investigation of stance, framing, and attribution.

## Footnotes

1 Previous attempts on such a reconciliation effort have been reported by Maslova (2015) and Cappelli (2006).

2 Kinneavy’s evaluation and Werlich’s argumentative text type overlap with behavioural and expository types in Longacre’s schema Virtanen (1992a).

3 COVID-19 Research Aspect Dataset (CODA-19): http://CODA-19.org.

4 The first version of the dataset (CTD v1.0) contains only contemporary news and historical travel reports for a total of 109 texts (Sprugnoli et al., 2017a).

5 https://en.wikisource.org/.

6 https://www.gutenberg.org/.

7 https://wikitravel.org/.

8 https://www.lonelyplanet.com/.

9 Every token in the clause is fully annotated including punctuation, and every clause is marked with one category.

10 Data and script used for calculating the IAA are available in the Github repository: https://github.com/johnnymoretti/ContentTypes.

11 In this setting, an error can correspond to a mismatch in the clause extent **and** in the assigned class or in the clause extent only.

12 https://github.com/UKPLab/elmo-bilstm-cnn-crf.

13 The values of the variational dropout are set to 0.3 both for the output dropout and the recurrent dropout.

14 The system described in the original CT paper could not be replicated because, as already mentioned in Sprugnoli et al. (2017a), it is not possible to properly extract features for all clauses, making a proper comparison with the bi-LSTM network impossible.

15 https://lindat.mff.cuni.cz/services/udpipe/.

16 https://taku910.github.io/crfpp/.

17 Note that we trained five different models and selected the best, similarly to the experiments with word embeddings described in Sect.  5.1.
